# Supplementary material for: Transcriptome analyses of 7-day-old zebrafish larvae possessing a familial Alzheimer’s disease-like mutation in psen1 indicate effects on oxidative phosphorylation, ECM and MCM functions, and iron homeostasis
Source: BMC Genomics. 2021 Mar 24;22:211. doi: 10.1186/s12864-021-07509-1 (PMC7992352; doi:10.1186/s12864-021-07509-1)
Supplement: Supplementary file 1 — Additional file 1: Supplementary data 1. Testing for differences in cell type proportions between genotypes. [file 12864_2021_7509_MOESM1_ESM.docx]

**Supplementary data 1: Testing for differences in cell type proportions between genotypes**

Apparent changes to gene expression could be due artefactual in nature due to differences in cell type proportions within the zebrafish larvae samples. To assess whether this is the case in the Q96_K97del/+ larvae, we first obtained gene sets describing genes which are expressed within various cell types/tissues in zebrafish larval cell types from [1]. We restricted gene sets to only include those which contained more than 10 genes.

To test for whether the genes within the marker gene sets tended to be differentially expressed, we used *mroast* [2] from the *limma* [3] package. No gene sets were found to contain a significant number of up or downregulated genes after correction for multiple testing using the false-discovery rate (directional p-value).

| Gene set | Total number of genes in gene set | Number of genes with increased expression  z > √2 | Number of genes with decreased expression  z < -√2 | Number of genes with no change to expression | Overall direction of change | p-value | FDR |
| --- | --- | --- | --- | --- | --- | --- | --- |
| Integument | 120 | 4 | 47 | 69 | Down | 0.0095 | 0.19 |
| Lens placode | 24 | 13 | 0 | 11 | Up | 0.044 | 0.3 |
| Visceral mesoderm | 22 | 1 | 7 | 14 | Down | 0.083 | 0.3 |
| Muscle | 37 | 7 | 10 | 20 | Down | 0.084 | 0.3 |
| Paraxial mesoderm | 30 | 3 | 8 | 19 | Down | 0.1 | 0.3 |
| Placode | 60 | 8 | 15 | 37 | Down | 0.1 | 0.3 |
| Pancreas | 23 | 7 | 2 | 14 | Up | 0.11 | 0.3 |
| Neural crest | 122 | 6 | 47 | 69 | Down | 0.11 | 0.3 |
| Thymus | 13 | 0 | 5 | 8 | Down | 0.18 | 0.42 |
| Liver | 31 | 3 | 9 | 19 | Down | 0.35 | 0.73 |
| Central Nervous System | 322 | 53 | 34 | 235 | Up | 0.52 | 0.8 |
| Pectoral fin bud | 27 | 3 | 4 | 20 | Down | 0.55 | 0.8 |
| Notochord | 11 | 0 | 2 | 9 | Down | 0.56 | 0.8 |
| Neuron | 28 | 3 | 4 | 21 | Down | 0.58 | 0.8 |
| Blood vessel | 23 | 3 | 3 | 17 | Up | 0.65 | 0.8 |
| Lateral plate mesoderm | 15 | 3 | 1 | 11 | Up | 0.67 | 0.8 |
| Kidney | 14 | 3 | 3 | 8 | Down | 0.68 | 0.8 |
| Retina | 27 | 6 | 6 | 15 | Down | 0.7 | 0.8 |
| Intestine | 45 | 1 | 4 | 40 | Down | 0.72 | 0.8 |
| Mesenchyme-related, organ muscle | 14 | 3 | 3 | 8 | Up | 0.8 | 0.81 |
| Blood | 14 | 1 | 2 | 11 | Down | 0.81 | 0.81 |

Nevertheless, we inspected the changes to gene expression in the top two most significantly altered marker gene sets: *Integument* and *Lens placode.* The overall distribution of the gene expression values is similar across genotypes.


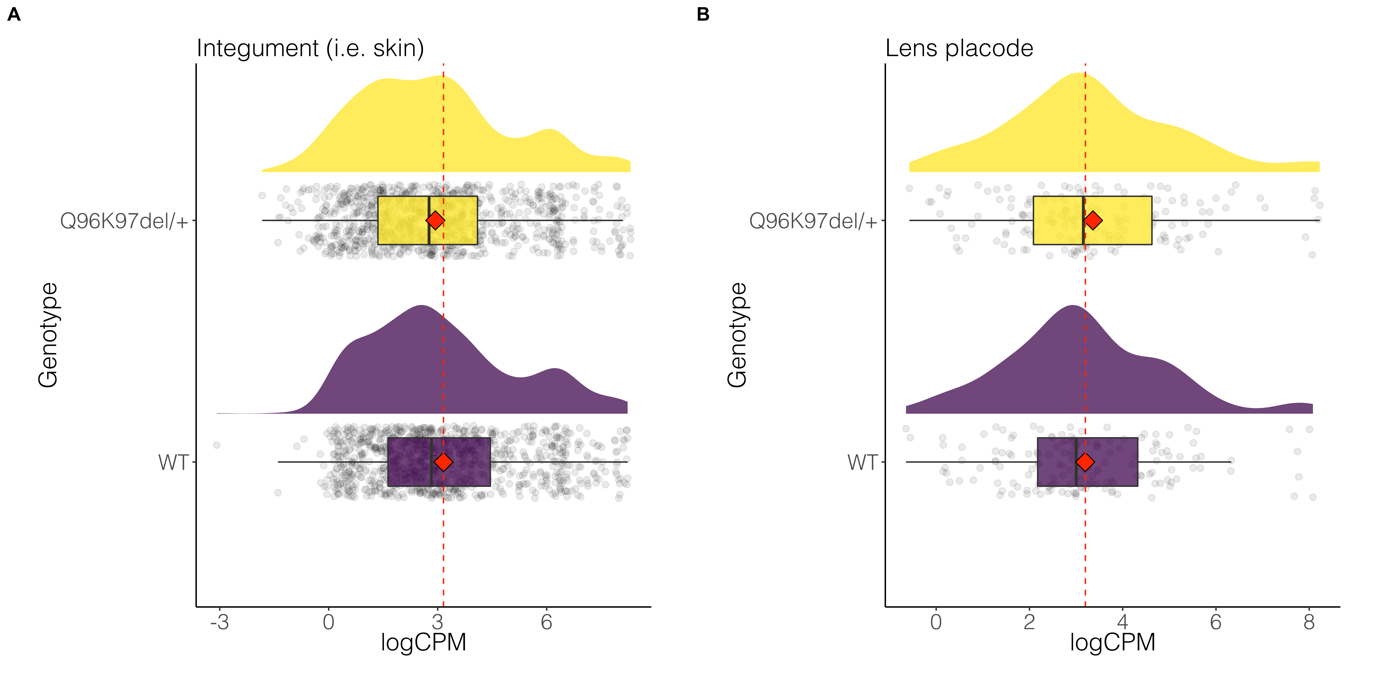


Raincloud plots showing the distribution of marker genes of larval zebrafish A) integument and B) Lens placode. Individual gene expression values are shown by the black points, and are summarised by boxplots and violin plots. The mean of the expression values are shown by the red diamond, while the red dashed line indicates specifically the mean in wild type (WT) samples.

Heatmap indicating the expression (logCPM) values of marker genes of larval zebrafish *integument*. Expression values are clustered based on their Euclidean distance.

Heatmap indicating the expression (logCPM) values of marker genes of larval zebrafish *Lens placode*. Expression values are clustered based on their Euclidean distance.

In summary, it does not appear that the changes to gene expression due to *psen1* genotype are due to changes in cell type proportions.

**References**

1. Farnsworth DR, Saunders LM, Miller AC: A single-cell transcriptome atlas for zebrafish development. *Developmental Biology* 2020, 459(2):100-108.

2. Wu D, Lim E, Vaillant F, Asselin-Labat M-L, Visvader JE, Smyth GK: ROAST: rotation gene set tests for complex microarray experiments. *Bioinformatics* 2010, 26(17):2176-2182.

3. Ritchie ME, Phipson B, Wu D, Hu Y, Law CW, Shi W, Smyth GK: limma powers differential expression analyses for RNA-sequencing and microarray studies. *Nucleic Acids Research* 2015, 43(7):e47-e47.
