# Supplementary material for: Transcriptome analyses of 7-day-old zebrafish larvae possessing a familial Alzheimer’s disease-like mutation in psen1 indicate effects on oxidative phosphorylation, ECM and MCM functions, and iron homeostasis
Source: BMC Genomics. 2021 Mar 24;22:211. doi: 10.1186/s12864-021-07509-1 (PMC7992352; doi:10.1186/s12864-021-07509-1)
Supplement: Supplementary file 5 — Additional file 5: Supplementary data 5. KEGG pathway diagrams. Diagrams for 6 significantly changed KEGG pathways not shown in the main manuscript. [file 12864_2021_7509_MOESM5_ESM.docx]

**Supplementary data 5: KEGG pathway diagrams**

Ten significantly-changed Kyoto Encyclopaedia of Genes and Genomes (KEGG) pathways were identified by GSEA. The KEGG diagrams of *DNA replication, cell cycle, ECM receptor interaction* and *oxidative phosphorylation* are shown in the main paper. The diagrams of the other 6 significantly changed KEGG pathways are listed below. Intensity of colour in diagrams represents gene log_2_FC ranging from -0.8 to 0.8 with downregulation in green and upregulation in red. Pathway maps are displayed with copyright permission from KEGG.

**Focal adhesion**

**
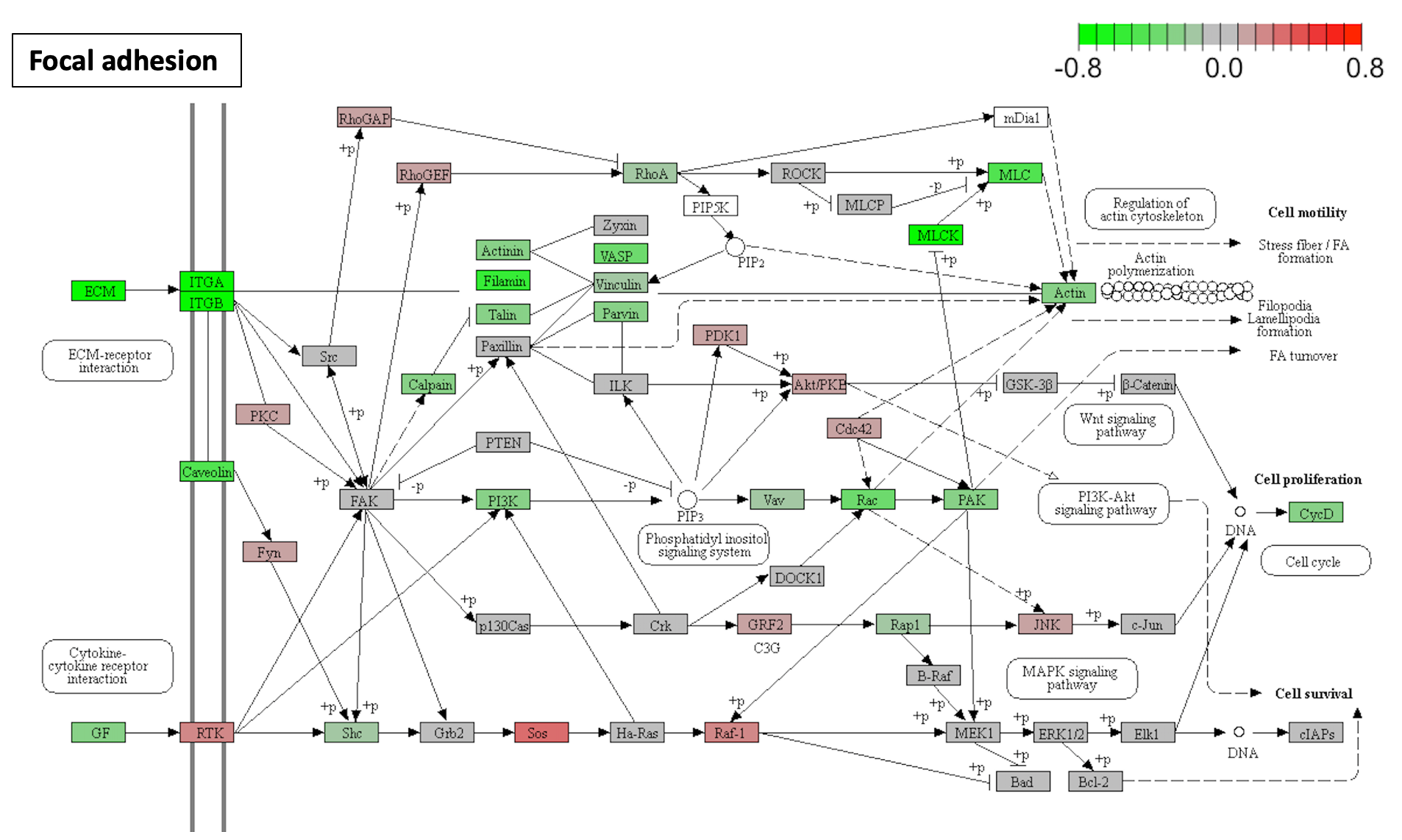
**

**Fatty acid metabolism**

**
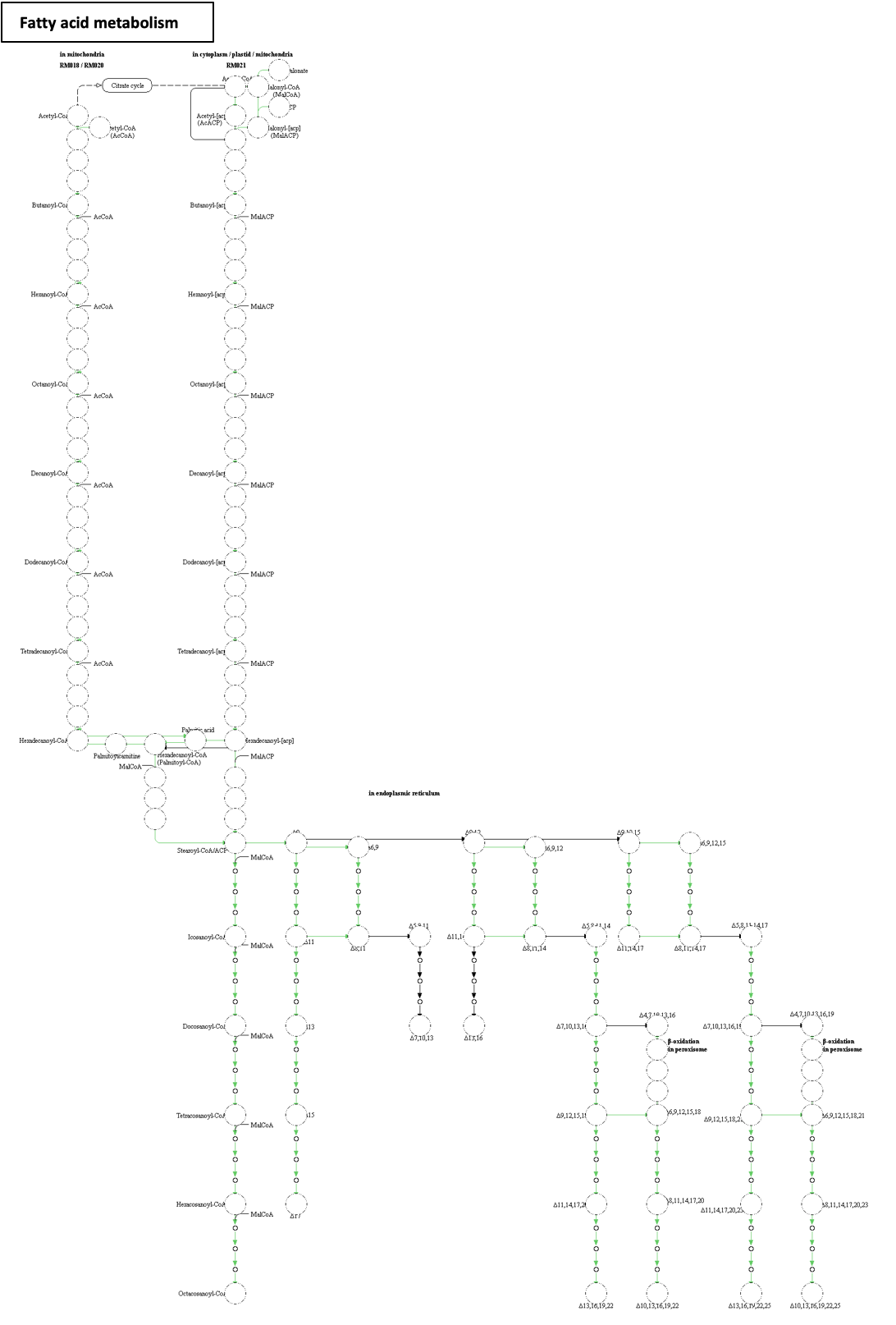
**

**Beta-Alanine metabolism**

**
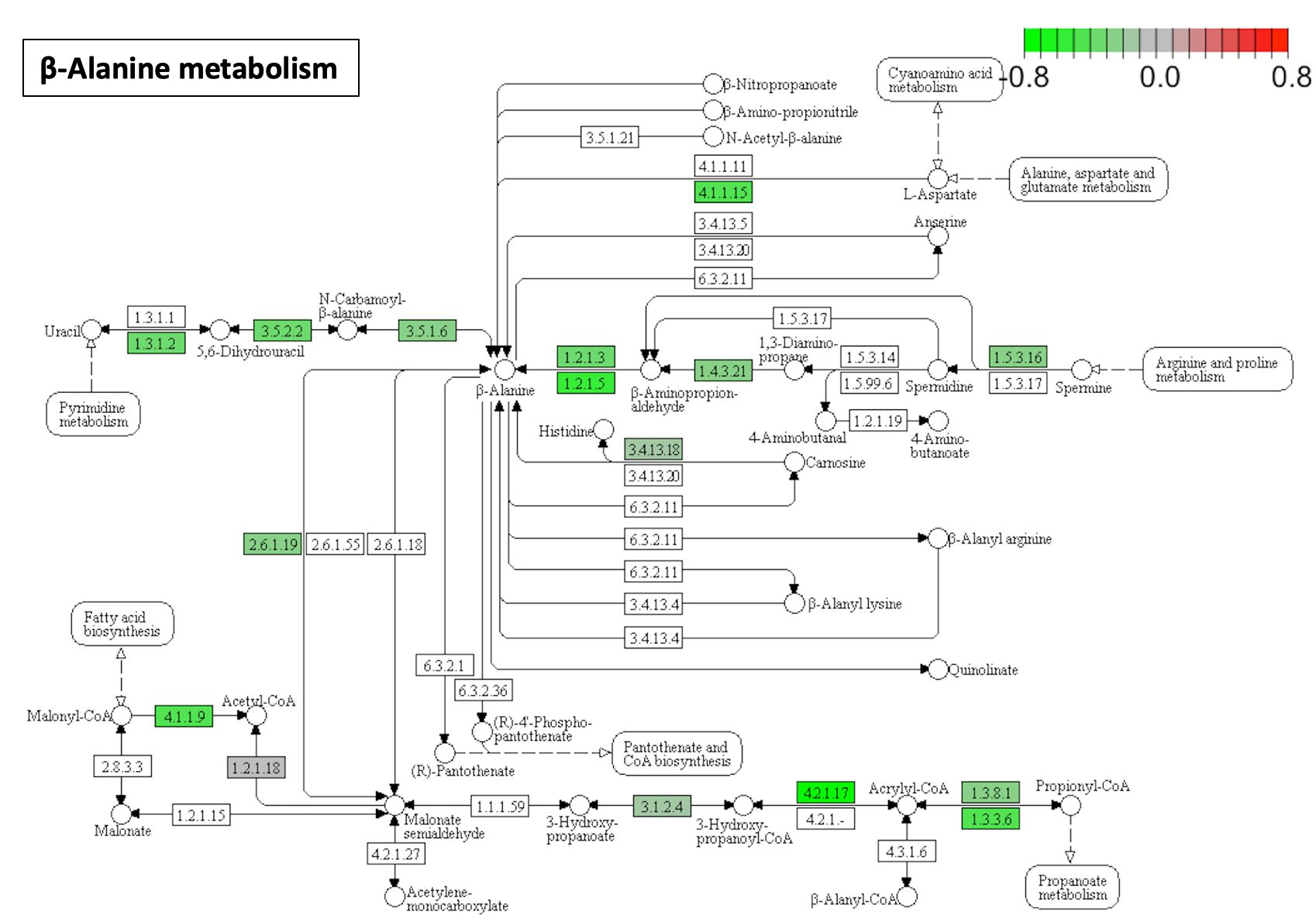
**

**Glutathione metabolism**

**
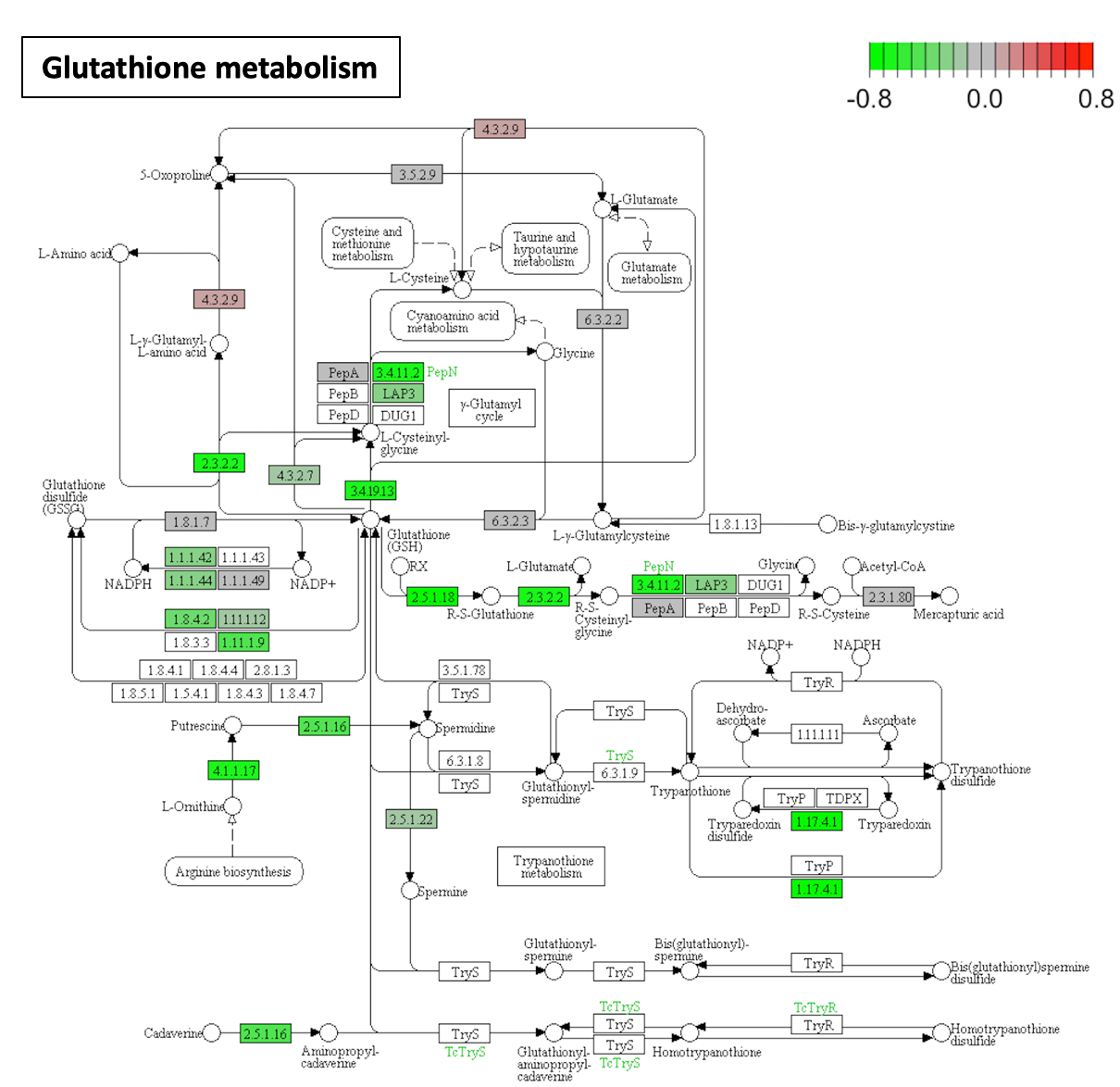
**

**Pyrimidine metabolism**

**
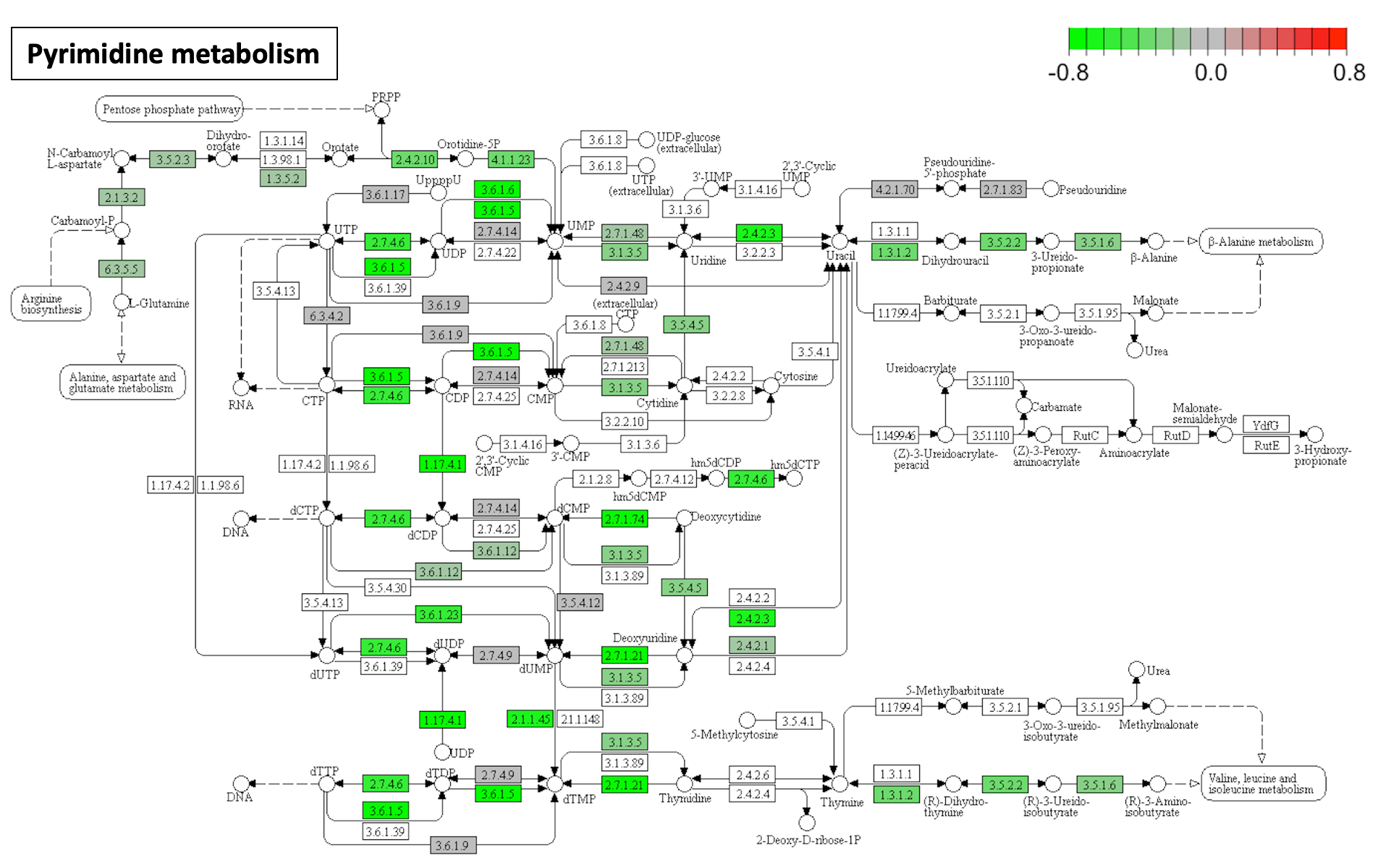
**

**Butanoate metabolism**

**
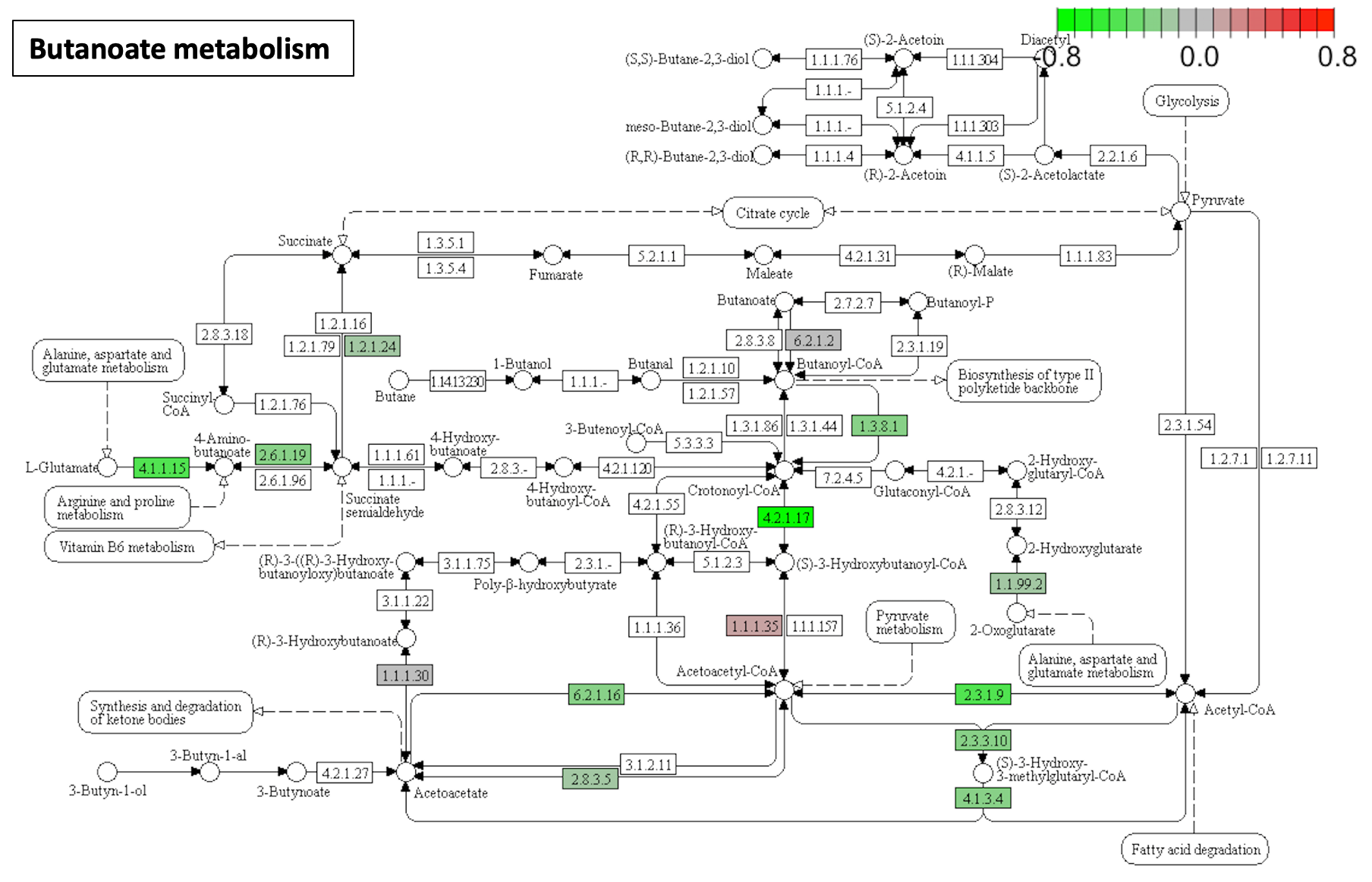
**
