# Supplementary material for: Transcriptome analyses of 7-day-old zebrafish larvae possessing a familial Alzheimer’s disease-like mutation in psen1 indicate effects on oxidative phosphorylation, ECM and MCM functions, and iron homeostasis
Source: BMC Genomics. 2021 Mar 24;22:211. doi: 10.1186/s12864-021-07509-1 (PMC7992352; doi:10.1186/s12864-021-07509-1)
Supplement: Supplementary file 6 — Additional file 6: Supplementary data 6. Weighted gene co-expression network analysis (WGCNA). [file 12864_2021_7509_MOESM6_ESM.docx]

**Supplementary data 6: Weighted gene co-expression network analysis (WGCNA)**

Weighted gene co-expression network analysis (WGCNA) is a systems biology method for studying biological networks based on the relationships between clusters of highly-correlated genes and variables [1]. Here, we performed WGCNA on our transcriptome data to explore transcriptomic changes at a systems level. This could define clusters of correlated genes with altered regulation due to the *psen1^Q96_K97del^* mutation.

**Method**

**WGCNA**

WGCNA [1] was performed on highly-expressed genes (those receiving more than 1.5 counts per million reads (CPM) in at least 6 samples) with sufficiently high variation (above 75% standard deviation across all samples). 4144 genes were retained in this analysis. A soft thresholding power of 13 was achieved by calculating the signed Pearson correlation for the construction of high scale-free weighted networks. This was then used to produce a signed adjacency matrix. Next, the adjacency matrix was transformed into a signed topological overlap matrix (TOM) for measurement of node similarity. A hierarchical clustering dendrogram was generated based on the dissimilarity (1-TOM) as the distance measure. The Hybrid Tree Cut method from dynamicTreeCut [2] was used to identify modules of co-expressed genes using the following parameters: 80 as minimum cluster size, 0.25 as minimum gap, 0.75 as maximum core scatter, using Partitioning Around Medoids (PAM) and 0.8 as cut height. The module eigengenes (MEs) were calculated using the gene sets in each module, and were then correlated to traits (pair and genotype). The correlations between modules and traits were plotted using pheatmap [3]. The gene ontology (GO) enrichment analysis on each module was performed using anRichment package [4], and a Bonferroni-corrected p-value < 0.05 was used to identify significant enriched GO terms. Also, for modules having p-value < 0.05, the genes in those modules were imported into STRING [5] for enrichment analysis with known databases (e.g. GO, KEGG) and visualisation of known biological relationships between genes.


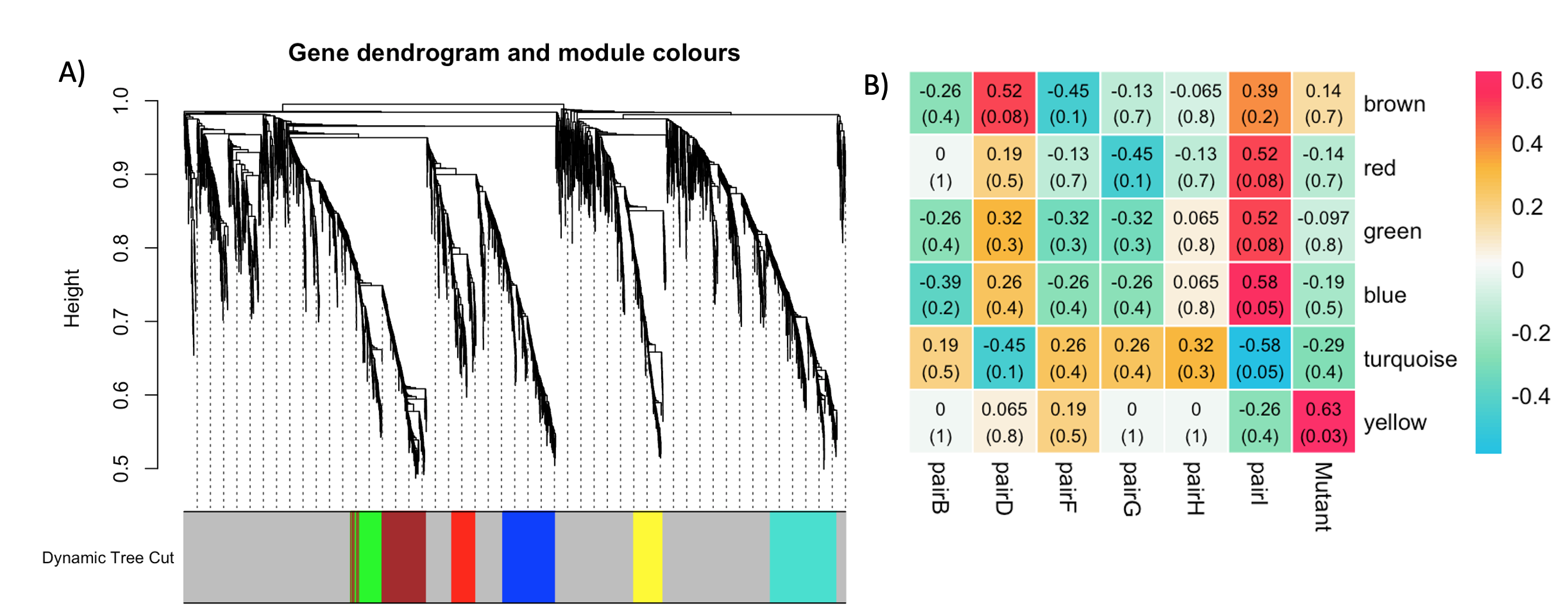


A) Gene hierarchical clustering dendrogram with modules of genes identified using dynamic tree cut. B) heatmap showing correlation between modules and traits with p-values shown in brackets.

6 modules of genes were identified using dynamic tree cut (Figure A). The correlations between modules and traits (pair and genotype) are shown in a heatmap (Figure B). Only the yellow module is significantly correlated with mutant genotype (Pearson correlation p-value < 0.05). GO enrichment analysis on the yellow module of genes identified two significantly enriched GO terms as listed in the following table. These two GO terms are not very informative, and do not show correlation with the results of other analyses. The enrichment analysis performed on the genes in the yellow module using STRING [5] did not identify any significantly enriched GO term or KEGG pathway.

| Module | GO ID | GO term | Bonferroni-corrected p-value |
| --- | --- | --- | --- |
| Yellow | GO:0009416 | response to light stimulus | 0.00374158 |
|  | GO:0009648 | photoperiodism | 0.02403991 |

In conclusion, the WGCNA performed here did not identify informative networks. The major limitation of our analysis is likely that the sample size (12 samples) does not meet the recommended minimum sample size (15 samples) for WGCNA [1]. Correlation on fewer than 15 samples can be too noisy for investigation of biologically meaningful networks.

**References**

1. Langfelder, P. and S. Horvath, *WGCNA: an R package for weighted correlation network analysis.* BMC Bioinformatics, 2008. **9**: p. 559.

2. Langfelder, P., B. Zhang, and S. Horvath, *Defining clusters from a hierarchical cluster tree: the Dynamic Tree Cut package for R.* Bioinformatics, 2008. **24**(5): p. 719-20.

3. Kolde, R., *pheatmap: Pretty Heatmaps.* 2019.

4. Langfelder, P., *anRichment: Collections and annotation data for use with anRichmentMethods.* 2020.

5. Szklarczyk, D., et al., *STRING v11: protein-protein association networks with increased coverage, supporting functional discovery in genome-wide experimental datasets.* Nucleic Acids Res, 2019. **47**(D1): p. D607-D613.
